# A Single Amino Acid Switch in the Adenoviral DNA Binding Protein Abrogates Replication Center Formation and Productive Viral Infection

**DOI:** 10.1128/mbio.00144-22

**Published:** 2022-03-07

**Authors:** Jana Boddin, Wing-Hang Ip, Britta Wilkens, Konstantin von Stromberg, Wilhelm Ching, Emre Koyuncu, Luca D. Bertzbach, Thomas Dobner

**Affiliations:** a Department of Viral Transformation, Leibniz Institute for Experimental Virology (HPI), Hamburg, Germany; Princeton University

**Keywords:** human adenovirus (HAdV), DNA binding protein (DBP), cellular ubiquitin-specific protease 7 (USP7), virus replication, viral gene expression, viral replication compartments (RC), replication-deficient mutant, adenoviral vector refinement

## Abstract

Adenoviruses are very efficient high-capacity vaccine vectors and are common gene delivery systems. Despite their extensive use in preclinical models and clinical trials over the past decades, adenoviral vectors still require optimization. To achieve that, more thorough characterizations of adenoviral genes and gene products, as well as pathogen-host interactions, are indispensable. The adenoviral DNA binding protein (DBP) is a key regulatory protein involved in various cellular and viral processes. Here, we show that single amino acid exchange mutations in human adenovirus C5 (HAdV-C5) DBP strongly influence adenoviral replication by altering interaction with the cellular ubiquitination machinery. Specifically, phenotypic analyses of DBP mutants demonstrate that single amino acid substitutions can regulate interactions with the cellular USP7 deubiquitinase, impede viral DNA synthesis, and completely abolish viral late protein expression and progeny production. Importantly, cells infected with the DBP mutant UBM5 consistently lack DBP-positive replication centers (RCs), which are usually formed during the transition from the early to the late phase of infection. Our findings demonstrate that DBP regulates a key step at the onset of the late phase of infection and that this activity is unambiguously linked to the formation and integrity of viral RCs. These data provide the experimental basis for future work that targets DBP and its interference with the formation of viral RCs during productive infection. Consequently, this work will have immediate impact on DNA virus and adenovirus research in general and, potentially, also on safety optimization of existing and development of novel adenoviral vectors and anti-adenoviral compounds.

## INTRODUCTION

Human adenovirus (HAdV) infection is a health concern with high prevalence worldwide (1–4). While HAdV infections are usually asymptomatic and self-limiting, they can cause serious upper and lower respiratory tract diseases and affect the gastrointestinal tract or the eyes in younger patients (5–13). HAdVs can cause severe infection with fatal consequences in immunocompromised patients such as hematopoietic stem cell- or organ transplant recipients (14–17). To date, we lack specific and effective treatment options for HAdV infections. Importantly, adenoviral vectors are among the most efficient gene delivery systems and are widely used in vaccine and gene therapy applications. However, adenoviral vectors still require optimization, as safety concerns, including severe side effects and vector persistence, remain (18).

The HAdV genome is a linear, double-stranded DNA (dsDNA) genome of approximately 36 kb that is organized in immediate early, early, intermediate, and late transcription units (19). The onset of viral DNA replication marks the transition from the early to the late phase of infection (20, 21). A key adenoviral protein that is indispensable for the DNA replication process is the DNA binding protein (DBP), one of the viral early region 2 (E2) transcription unit proteins (20, 22). The HAdV serotype 5 (HAdV-C5) DBP is a 529-amino-acid product of the E2A gene and is expressed early and late in infection, regulated by different promoters (20, 23–25). At early time points postinfection, DBP diffusely localizes in the nucleus, condenses into small foci toward the end of the early stage of infection, and accumulates in spherical liquid biomolecular condensates during the late phase of infection (20, 23, 24, 26, 27). These membrane-less structures provide a hub for several viral and cellular proteins and correspond to viral replication centers (RCs), which are not only hot spots for viral DNA replication, late gene expression, and virus assembly but also for the sequestration and inactivation of host restriction factors and the recruitment of proviral factors that facilitate efficient viral transcription (26, 28–31). However, viral RC formation and their composition, as well as the contribution of some viral and especially cellular factors localizing at these sites, remain elusive. Apart from its major role in DNA replication, DBP is also involved in transcriptional control, mRNA stability, virus assembly, viral transformation, and host range determination (32–42).

Previously, we identified that the cellular ubiquitin-specific protease 7 (USP7), a deubiquitinating enzyme, localizes in viral RCs after HAdV-C5 infection (43). Ubiquitin conjugation to target proteins impacts their function by regulating protein-protein interactions and localization as well as protein turnover (44). USP7 acts as a proviral factor, as USP7 inhibition by HBX41108 or protein knockdown reduces viral replication and leads to decreased levels of the viral early protein E1B-55K (43). In line with HAdV-C5, USP7 may provide functions in virus-infected cells, as it interacts with viral regulatory proteins, including Tat (human immunodeficiency virus [HIV-1]), EBNA1 (Epstein-Barr virus [EBV]), ICP0 (herpes simplex virus 1 [HSV-1]), LT (Merkel cell polyomavirus [MCPyV]), UL35 (cytomegalovirus [CMV]), LANA (Kaposi sarcoma-associated herpesvirus [KSHV]), as well as with LANA homologues from two other gamma-2 herpesviruses, murine gammaherpesvirus 68 and rhesus rhadinovirus (45–50). Also, USP7 plays important roles in various cellular processes, including cell division, apoptosis, tumorigenesis, and epigenetic regulation (51–59). Relocalization of USP7 to the RCs is independent of the adenoviral E1B-55K protein (43) but is likely associated with interactions with other viral components of RCs, most prominently DBP. To test this hypothesis, we investigated putative DBP-USP7 binding mutants and whether recruitment of USP7 to RCs is regulated through complex formation with DBP. Moreover, we examined whether the proviral functions of USP7 are dependent on its interaction with DBP and DBP-stimulated accumulation in RCs.

In this report, we show that HAdV-C5 DBP can bind to USP7 and that this interaction has an impact on its relocalization, sequestration, and accumulation into viral RCs. We identified a single amino acid mutation in the DBP C terminus that retains the ability of the protein to bind to USP7. Strikingly, this mutated DBP is highly ubiquitinated and renders the virus completely replication defective. Our results unequivocally demonstrate that RC formation is dependent on DBP and, remarkably, essential for progression into the late phase of infection, which is followed by viral progeny production. Our findings therefore provide important information on HAdV-C5 DBP that will also push antiadenoviral drug development and research on other pro- and metaphylactic antiviral therapies, especially regarding the use of HAdV as vectors in vaccination and gene therapy.

## RESULTS

### HAdV-C5 DBP binds to the TRAF-like domain of USP7.

**FIG 1:**
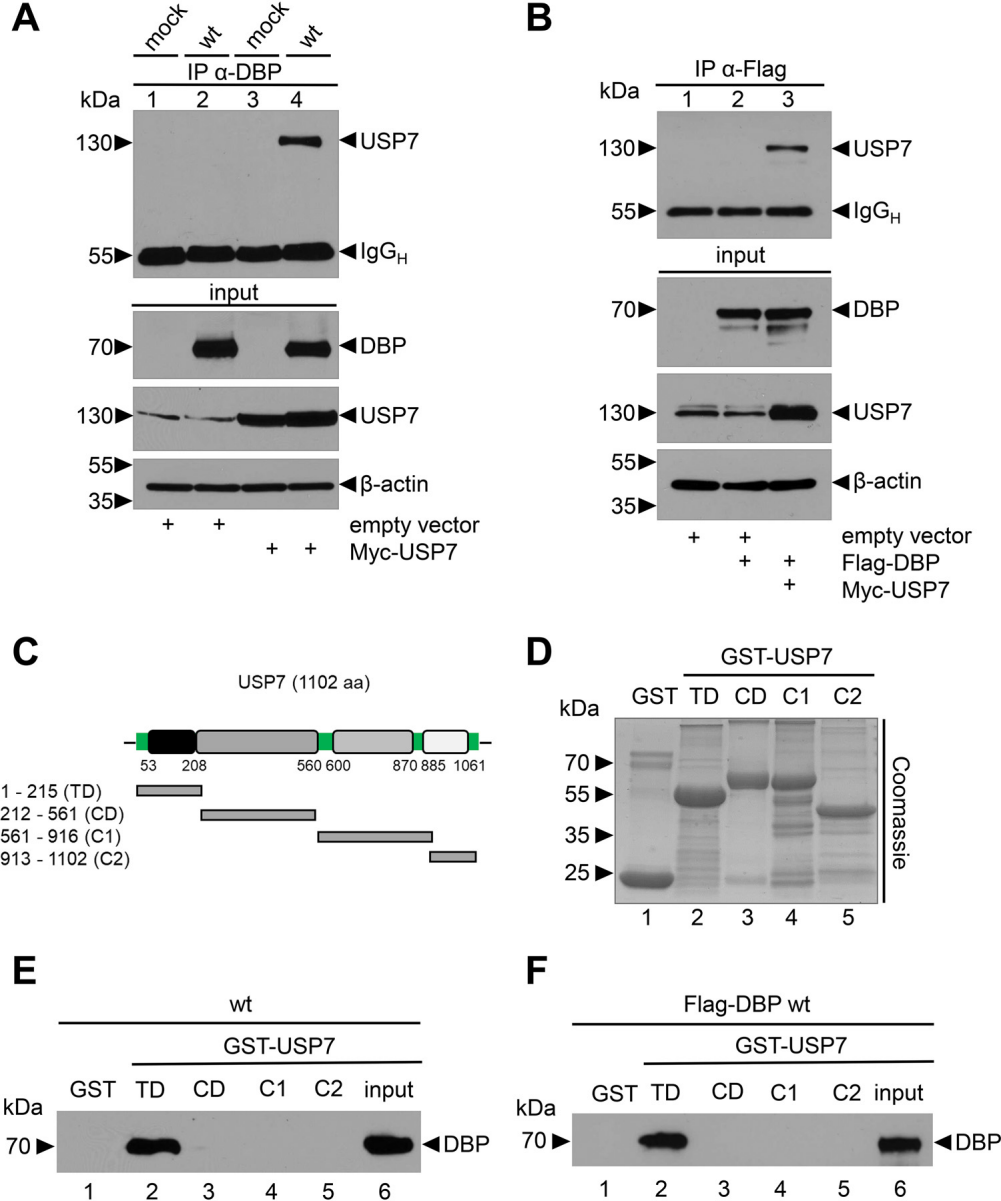
DBP binds to the TRAF-like domain of USP7. (A) Myc-USP7-transfected H1299 cells were mock infected or WT HAdV-C5 (H5*pg*4100) infected 24 hpt at an MOI of 20 focus-forming unit (FFU) per cell and harvested 48 h later. (B) H1299 cells were transfected with an empty vector control, Flag-DBP, or Myc-USP7 plasmids and harvested at 48 hpt. Total cell lysates were prepared, DBP was immunoprecipitated with MAb B6-8 (α-DBP) (A) or MAb Flag-M2 (α-Flag) (B), and proteins were resolved by 10% SDS-PAGE and visualized by immunoblotting. Coprecipitated proteins and total cell lysates (input) were analyzed using an α-USP7 antibody. An α-DBP antibody was used to stain DBP in total cell lysates, and β-actin served as a loading control. Molecular weights (in kDa) are indicated to the left and detected proteins to the right of the blots. Detailed antibody descriptions can be found in the respective Materials and Methods paragraphs. (C) Schematic representation of USP7 with its N-terminal TRAF-like domain (TD), catalytic domain (CD), and C-terminal structural domains C1 and C2 (modified from reference 43). (D) Coomassie-stained GST-USP7 fusion constructs. (E and F) GST-USP7 pulldowns with WT H5*pg*4100-infected (E) and FLAG-DBP-transfected (F) H1299 cell lysates using the indicated different USP7 constructs.

It has previously been shown that DBP colocalizes with USP7 in viral RCs, independent of its viral interaction partner E1B-55K. Furthermore, short hairpin RNA (shRNA)-mediated depletion of USP7 or inhibition by the USP7 inhibitor HBX substantially reduces steady-state concentrations of DBP in wild-type (WT) H5*pg*4100-infected cells (43), suggesting that DBP interacts with and might be deubiquitinated by USP7 and that this activity may direct the host protein into RCs. To test this hypothesis, we performed protein binding assays with virus-infected and plasmid-transfected H1299 cells (60) and HCT116 cells (61) (Fig. 1). Combined immunoprecipitation/immunoblotting experiments show that DBP specifically interacts with USP7 in WT H5*pg*4100-infected H1299 cells upon USP7 overexpression (Fig. 1A) or with endogenous USP7 in infection experiments of HCT116 cells (Fig. S1 in the supplemental material). Identical results were obtained with plasmid-transfected H1229 cells expressing epitope-tagged DBP and USP7 fusion proteins only (Fig. 1B). To map the region in USP7 responsible for binding to DBP, we performed pulldown assays with glutathione *S*-transferase (GST) fusion proteins containing different segments of USP7, including its N-terminal TRAF (tumor necrosis factor receptor [TNFR]-associated factor)-like domain (TD), a central catalytic domain (CD), and two C-terminal ubiquitin-like (UBL) domains (C1 and C2) (62–64) (Fig. 1C to F). Among those, only the N-terminal segment of USP7 precipitated DBP from virus-infected cell lysates (Fig. 1E) or plasmid-transfected H1299 cells expressing epitope-tagged DBP (Fig. 1F). Altogether, these data demonstrate that USP7 can bind to DBP independently of other viral proteins. Moreover, complex formation between both proteins involves the TRAF-like domain of USP7, previously shown to mediate the interaction with USP7 binding partners, such as p53 and MDM2 (63, 65).

10.1128/mbio.00144-22.1 FIG S1 Endogenous USP7 binds DBP in virus-infected HCT116 cells. Empty vector- or Myc-USP7-transfected HCT116 cells were mock infected or WT HAdV-C5 (H5*pg*4100) infected 24 hpt at an MOI of 20 FFU per cell and harvested 48 h later. Total cell lysates were prepared, DBP was immunoprecipitated using an α-DBP antibody, and proteins were resolved by 10% SDS-PAGE and visualized by immunoblotting. Coprecipitated proteins and total cell lysates (input) were analyzed using an α-USP7 antibody. An α-DBP antibody was used to stain DBP in total cell lysates and β-actin served as a loading control. Molecular weights (in kDa) are indicated to the left and detected proteins to the right of the blots. Detailed antibody descriptions can be found in the respective Materials and Methods paragraphs. Download FIG S1, PDF file, 0.2 MB. Copyright © 2022 Boddin et al. 2022 Boddin et al. https://creativecommons.org/licenses/by/4.0/ This content is distributed under the terms of the Creative Commons Attribution 4.0 International license.

### A USP7 binding motif in the amino-terminal region of DBP mediates the interaction with USP7.

**FIG 2:**
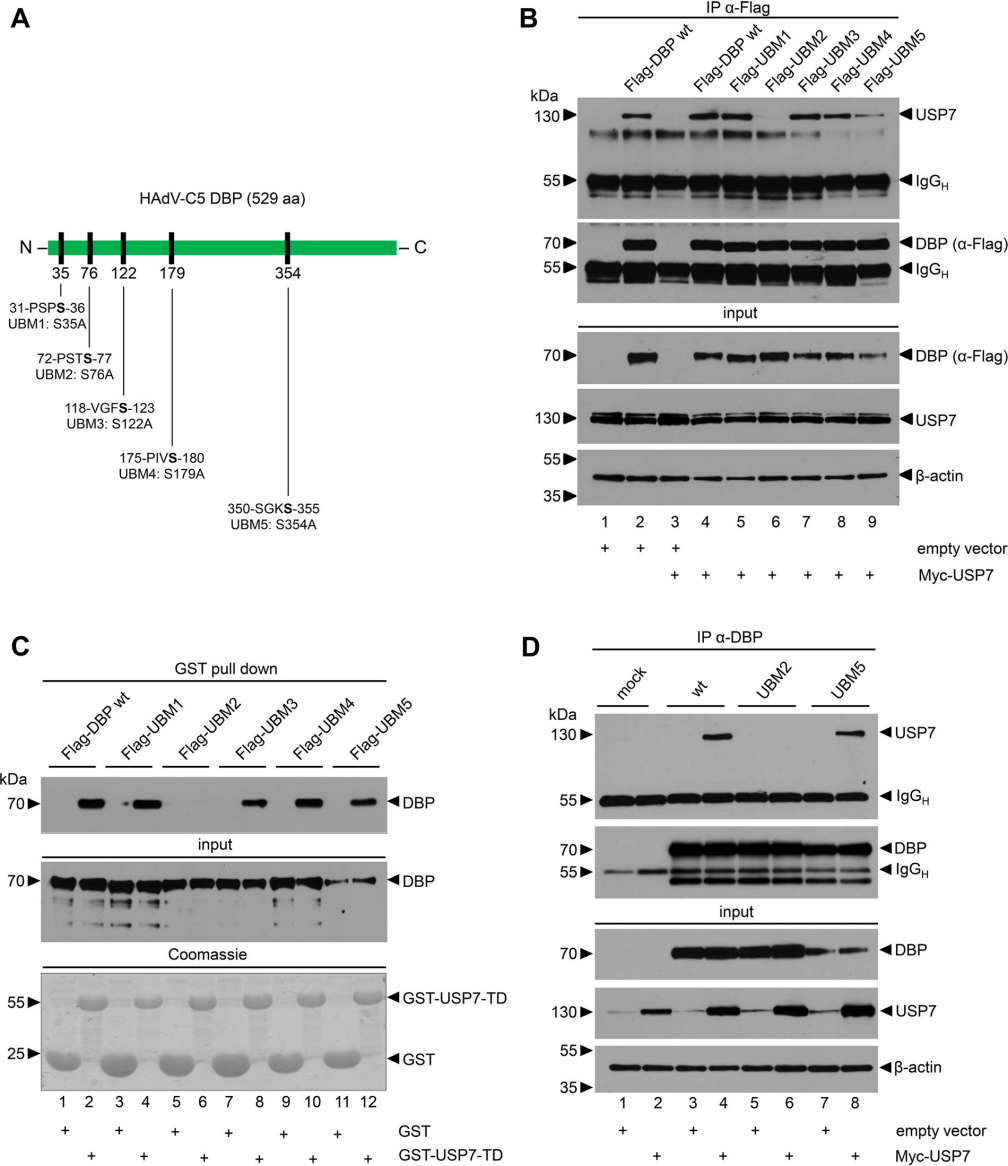
The S76A substitution in UBM2 abrogates binding to USP7. (A) Schematic representation of HAdV-C5 DBP with the five potential USP7 binding sites. (B) HCT116 cells were transfected with an empty vector control, Myc-USP7, or the respective Flag-DBP mutant plasmids and harvested at 48 hpt. Total cell lysates were prepared, DBP was immunoprecipitated with MAb Flag-M2 (α-Flag), and proteins were resolved by 10% SDS-PAGE and visualized by immunoblotting. Coprecipitated proteins and total cell lysates (input) were analyzed for USP7. β-Actin served as a loading control. Molecular weights (in kDa) are indicated to the left and detected proteins to the right of the blots. Detailed antibody descriptions can be found in the respective Materials and Methods paragraphs. (C) GST-USP7 pulldown of FLAG-DBP-transfected H1299 cell lysates using the different DBP constructs, including the Coomassie-stained control. (D) Myc-USP7-transfected H1299 cells were WT H5*pg*4100, UBM2 H5*pm*4250, and UBM5 H5*pm*4251 infected at 24 hpt (MOI of 20) and analyzed for DBP-USP7 coimmunoprecipitation in USP7-overexpressing cells at 48 hpi as described in panel A.

Previous studies have shown that binding to USP7 involves short four-amino-acid segments in the substrate proteins (e.g., p53, MDM2, and EBNA1) that resemble previously published consensus sequences (65, 66). In fact, five of these consensus motifs are also found present in HAdV-C5 DBP, located primarily in its N-terminal region (Fig. 2A). To analyze their role in binding to USP7, we substituted the last serine residue in each of the motifs with an alanine (Fig. 2A). The corresponding variants were designated USP7 binding mutants 1 to 5 (UBM1 to UBM5) (Fig. 2A). Subsequently, the interaction with USP7 was analyzed by combined immunoprecipitation/immunoblotting from cotransfected HCT116 cells expressing epitope-tagged versions of USP7 (Myc) and DBP WT or UBMs (Flag) (Fig. 2B). UBM1, -3, and -4 precipitated USP7 comparable to WT DBP (Fig. 2B, lanes 4, 5, 7, and 8). Less USP7 was detected in the precipitates with UBM5, likely due to the reduced steady-state levels of the mutant protein (Fig. 2B, lane 9). In contrast, no USP7 coprecipitated with UBM2 (Fig. 2B, lane 6), suggesting that the substitution of serine 76 with alanine (S76A) in the UBM2 motif abrogated the binding to USP7. To further verify the interaction between the USP7 TRAF-like domain and UBM2, we performed additional GST pulldown assays (Fig. 2C). We used the GST-fused USP7 TRAF-like domain and the DBP variants from plasmid-transfected cell lysates. Consistent with the USP7 immunoprecipitation experiments, only UBM2 failed to precipitate with the GST fusion protein containing the USP7 TRAF-like domain (Fig. 2C, lane 6). Finally, to reveal the effect of DBP mutations on USP7 binding in the context of virus-infected cells, we generated two HAdV-C5 mutants containing identical amino acid substitutions in the DBP UBM2 (H5*pm*4250) or UBM5 (H5*pm*4251) motifs. These were tested in plasmid-transfected and virus-infected H1299 cells (Fig. 2D). As expected, WT and UBM5 DBP coprecipitated similar amounts of epitope-tagged USP7, although the mutant protein accumulated to lower steady-state concentrations than WT DBP (Fig. 2D, lanes 4 and 8). Importantly, no USP7 coprecipitated with the UBM2 mutant (Fig. 2D, lane 6). Taken together, these data confirm that the UBM2 motif in DBP is necessary and sufficient for the interaction with the TRAF-like domain of USP7 *in vitro* and *in vivo*.

### UBM5 DBP is highly ubiquitinated.

**FIG 3:**
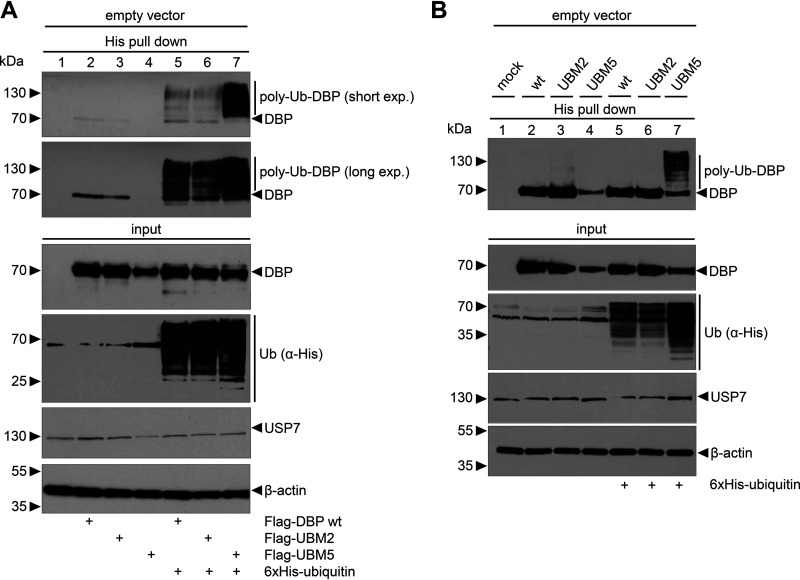
DBP is modified by ubiquitination resulting in high levels of polyubiquitinated UBM5 DBP. (A and B) His-ubiquitin-transfected H1299 cells were cotransfected with the different DBP mutant plasmids as indicated (A) or infected with WT H5*pg*4100 or the virus mutants UBM2 H5*pm*4250 and UBM5 H5*pm*4251 at an MOI of 10 (B) and subjected to His-ubiquitin pulldowns. Proteins were resolved by 10% SDS-PAGE and visualized by immunoblotting. Coprecipitated proteins and total cell lysates (input) were analyzed using α-DBP and α-His antibodies. An α-USP7 antibody was used to stain USP7 in total cell lysates. β-actin served as a loading control. Molecular weights (in kDa) are indicated to the left and detected proteins to the right of the blots. Detailed antibody descriptions can be found in the respective Materials and Methods paragraphs.

Ubiquitination can regulate the activity, function, or localization of a protein but can also lead to reduced protein stability and proteasomal degradation. Because USP7 can deubiquitinate target proteins, we set to investigate if that is the case for DBP. To determine the role of USP7 in the context of HAdV-C5 infection, we analyzed whether the viral DBP is ubiquitinated. In the next step, we determined if the amino exchanges in UBM2 and UBM5 and thus, USP7 binding, affect the ubiquitination status of these proteins. His-ubiquitin-transfected H1299 cells were either cotransfected with DBP-expressing plasmids (WT, UBM2, and UBM5) or coinfected with WT H5*pg*4100 or the virus mutants UBM2 H5*pm*4250 and UBM5 H5*pm*4251 (Fig. 3). His-ubiquitin pulldown experiments revealed that all transfected DBPs (WT, UBM2, and UBM5) were ubiquitinated and that UBM5 ubiquitination was strongly increased compared to WT (Fig. 3A, lanes 5 to 7). In contrast to the transfection experiments, ubiquitination of WT DBP and UBM2 was undetectable in infections (Fig. 3B, lanes 5 and 6), but clearly detectable in infections with UBM5 H5*pm*4251 (Fig. 3B, lane 7). These data demonstrate that DBP is posttranslationally modified by ubiquitination. However, the data do not allow to draw firm conclusions on the deubiquitination of DBP by cellular USP7. Importantly, we observed an increased ubiquitination of the UBM5 mutant in all experiments.

### UBM5 leads to a complete abrogation of viral RC formation.

**FIG 4:**
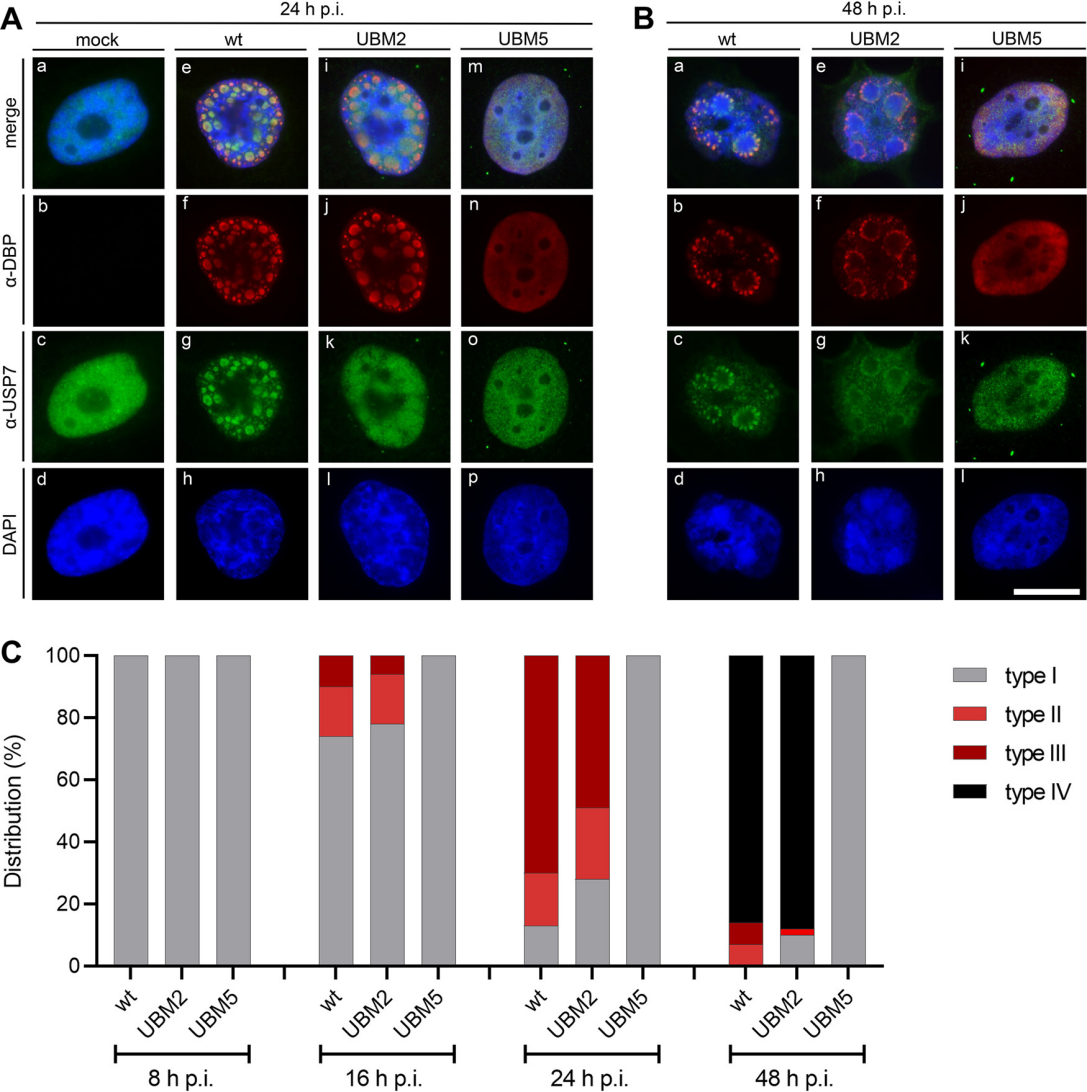
UBM2 abrogates USP7 relocalization to viral RCs, and UBM5 completely inhibits viral RC establishment. (A and B) Immunofluorescence analysis of WT H5*pg*4100-, UBM2 H5*pm*4250-, or UBM5 H5*pm*4251-infected H1299 cells after 4% PFA fixation at indicated time points (24 and 48 hpi). DBP (red), USP7 (green), and nuclei (blue) are visualized with secondary fluorescent antibodies that bind to the α-DBP mouse monoclonal antibody (MAb) B6-8 or the α-USP7 rat MAb 3D8 and DAPI, respectively. Representative images of *n* ≥ 70 analyzed cells for every virus infection. Scale bar represents 10 μm. (C) Time course analyses of the localization of the different DBPs revealed distinct patterns that we categorized into four types, I (diffuse), II (dot-like), III (classical replication compartments), and IV (ringlike structures) as previously reported (94). Immunofluorescence analyses were performed at 8, 16, 24, and 48 hpi, cells were counted and categorized, and percentages were calculated (an average of 70 cells were analyzed per infection and time point postinfection).

To explore the effect of the UBM2 and UBM5 mutations as well as the different ubiquitination levels on the accumulation of USP7 into RCs, the steady-state localization of USP7 was determined in infected H1299 cells and compared to WT DBP by double-label immunofluorescence at two different time points after infection. (Fig. 4). Consistent with our previous studies, USP7 was found diffusely distributed in the nucleus of noninfected cells (Fig. 4A, panels a to c). In contrast, at 24 h (Fig. 4A, panels e to g) and 48 h (Fig. 4B, panels a to c) after infection with WT H5*pg*4100, the intensity of diffuse nuclear staining was greatly reduced, and in all of the infected cells examined (*n* > 140), USP7 was clearly seen concentrated in DBP-positive RCs. While the nuclear distribution of USP7 was observed to be diffuse in UBM2 mutant H5*pm*4250-infected cells at 24 h postinfection (hpi) (Fig. 4A, panels i to k), it was comparable to WT infections at the later time point (48 hpi) (Fig. 4B, panels e to g). A completely different staining pattern was observed in cells infected with the UBM5 mutant H5*pm*4251. Surprisingly, none of the nuclei examined (*n* > 280) contained DBP-positive RCs at both time points postinfection (Fig. 4A, panels m to o, and Fig. 4B, panels i to k). Instead, the UBM5 mutant protein was seen diffusely distributed in the nucleus with the nucleoli excluded. Also, USP7 remained uniformly distributed in the nucleoplasm at 24 hpi, which changed to a more granular distribution at the later time point. Identical results were obtained in infected HCT116 cells (data not shown). Quantification of the different RC phenotypes at different time points postinfection confirmed the complete absence of RCs in UBM5 H5*pm*4251 infection and showed that the course of infection in WT H5*pg*4100- and UBM2 H5*pm*4250-infected cells resembled what has been previously reported, a temporal transition from diffuse DBP distribution within the nuclei to dot-like structures at 8 and 16 hpi, the presence of classical replication compartments at 24 hpi, and, eventually, “ringlike” structures, which are late, coalescing RCs (Fig. 4C).

Altogether, these data clearly show that the UBM2 mutation in DBP is neglectable for efficient recruitment of USP7 into RCs during late time points of a productive HAdV-C5 infection. Here, USP7 can accumulate at the periphery of viral RCs independently of DBP (Fig. 4) despite abrogated USP7 binding (Fig. 2). This presumably happens through functional interactions with other (cellular) USP7 interaction partners known to localize in RCs. Moreover, the observation that UBM5 H5*pm*4251-infected cells consistently lack DBP-positive RCs from the early to the late phase of a productive infection supports the idea that HAdV-C5 DBP controls the initiation of viral RCs and, thus, a key step at the onset of the late phase and that this activity is intimately linked to its ability to regulate the formation and/or integrity of viral RCs, including viral DNA replication.

### The single amino acid substitution in UBM5 impedes viral DNA synthesis and completely abolishes progeny production.

**FIG 5:**
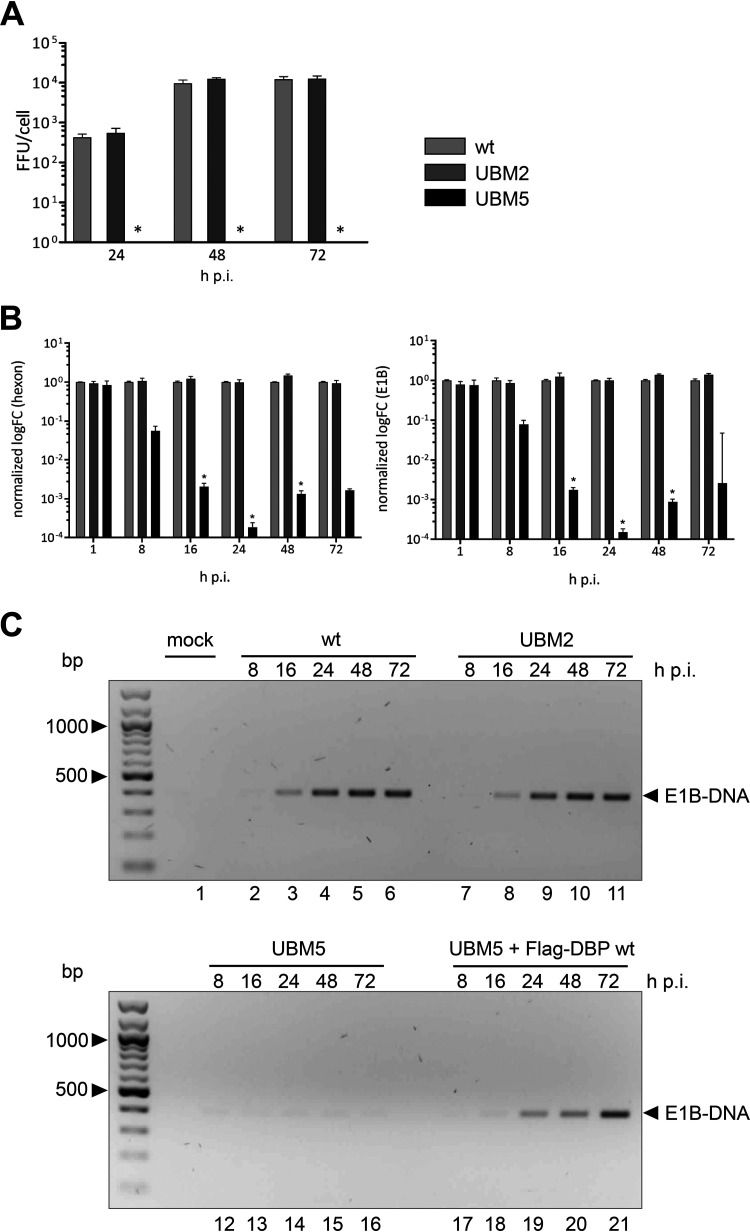
UBM5 is replication defective. (A) WT H5*pg*4100, UBM2 H5*pm*4250, and UBM5 H5*pm*4251 were used to assess viral progeny production in H1299 cells at 24, 48, and 72 hpi by virus titration as described previously (92). (B) qPCR analyses of hexon (left) or E1B DNA (right) in WT H5*pg*4100- (light gray), UBM2 H5*pm*4250- (dark gray), and UBM5 H5*pm*4251-infected H1299 cells (black) at different time points postinfection (*n* ≥ 3). Error bars indicate standard deviations. Asterisks indicate statistically significant differences of UBM5 H5*pm*4251 to WT H5*pm*4100 and UBM2 H5*pm*4250 (*, *P* < 0.05; one-way analysis of variance [ANOVA] with Bonferroni correction). (C) Viral DNA of WT H5*pg*4100-, UBM2 H5*pm*4250-, or UBM5 H5*pm*4251-infected H1299 cells and UBM5-infected/Flag-DBP WT-transfected H1299 cells were analyzed for E1B abundance by PCR at different time points postinfection.

To test this model, we determined total virus yield in H1299 cells infected with WT H5*pg*4100, UBM2 H5*pm*4250, and UBM5 H5*pm*4251 (Fig. 5). We found that the UBM5 mutant could only be produced in 2E2 cells that stably express WT HAdV-C5 DBP, suggesting that the mutation in this motif inactivates a function of DBP required for efficient virus replication. Replication of the UBM2 H5*pm*4250 mutant virus was comparable to that of WT H5*pg*4100 (Fig. 5A). Also, in line with this result, no differences were observed when we monitored viral DNA replication at different time points after the infection (Fig. 5B, lanes 2 to 6 and 7 to 11). However, as suspected, the UBM5 H5*pm*4251 mutant exhibited a severe defect in virus growth (Fig. 5A). Indeed, virus progeny production was totally abolished in UBM5 H5*pm*4251-infected cells. This phenotype directly correlated with a severe defect in viral DNA replication (Fig. 5B, lanes 12 to 16), which could be at least partially rescued by ectopic expression of Flag-tagged WT DBP (Fig. 5C). Identical results (not shown) were obtained from analyses using HCT116 cells.

In line with this result, DBP-positive aggregates were observed in the nuclei of these cells by double-label immunofluorescence (Fig. 4), again strongly suggesting that DBP-regulated formation of RCs is fundamental for efficient viral DNA replication and, as a consequence, for maximal late protein production.

### UBM5 completely abrogates late viral protein expression in HAdV-C5 infection.

**FIG 6:**
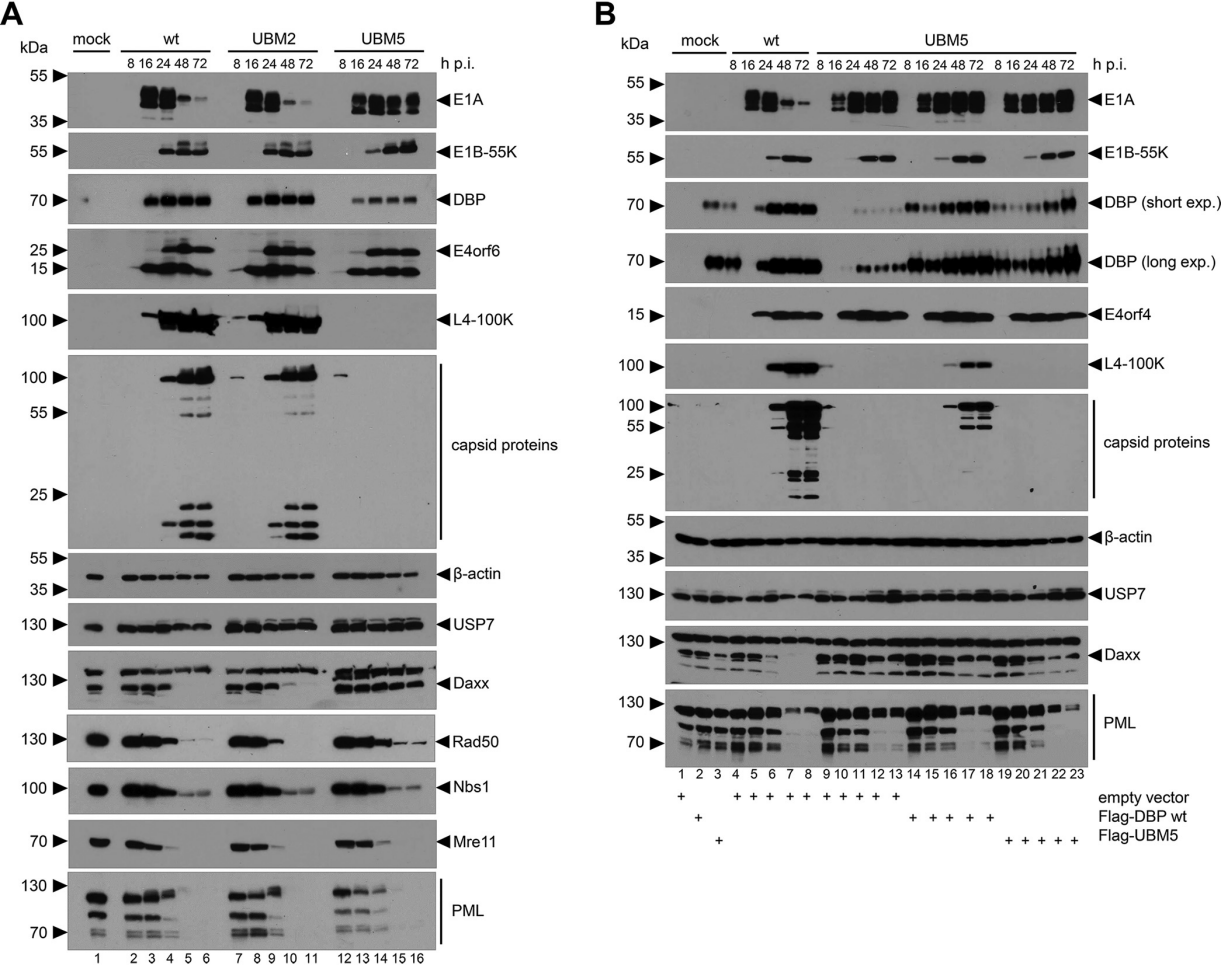
UBM5 shows impaired late viral protein expression. Immunoblotting of WT. (A and B) H5*pg*4100-, UBM2 H5*pm*4250-, and UBM5 H5*pm*4251-infected H1299 cells detecting indicated cellular and viral genes in plain infections (MOI of 20) (A) and DBP rescue infections (MOI of 10) (B) with Flag-DBP WT or UBM5 plasmids as indicated. Proteins were resolved by 10% SDS-PAGE and visualized by immunoblotting. Molecular weights (in kDa) are indicated to the left and detected proteins to the right of the blots. Cellular and viral proteins were detected with antibodies listed in Materials and Methods.

The onset of viral DNA replication is crucial for the expression of late viral genes and thereby induces the transition from the early into the late phase of infection (21). To further investigate the defective viral DNA synthesis of UBM5 H5*pm*4251, we analyzed the steady-state levels of a variety of early and late viral proteins in virus infections of H1299 cells by immunoblotting. Overall, levels of early and late proteins were similar in WT H5*pg*4100 or UBM2 H5*pm*4250-infected cells (Fig. 6A, lanes 2 to 6 and 7 to 11). However, we observed striking differences in early and late protein expression in UBM5 H5*pm*4251-infected cells compared to WT H5*pg*4100 (Fig. 6A, lanes 2 to 6 and 12 to 16). Interestingly, E1A, the first protein expressed during HAdV infection (67, 68), was expressed from 16 hpi during the entire infection cycle, along with E4orf6 and DBP in UBM5 H5*pm*4251-infected cells. In WT H5*pg*4100- and UBM2 H5*pm*4250-infected cells, E1A levels peaked at 16 hpi and decreased over time. Levels of other evaluated early proteins (E4orf6, E1B-55K) were comparable in WT H5*pg*4100-, UBM2 H5*pm*4250-, and UBM5 H5*pm*4251-infected cells. Strikingly, and in contrast to WT H5*pg*4100 and UBM2 H5*pm*4250, UBM5 H5*pm*4251 is entirely defective in late protein expression, as demonstrated for the late HAdV-C5 protein L4-100K and the capsid proteins (Fig. 6A, lanes 12 to 16). Thus, as a consequence of defective DNA replication (Fig. 5), the UBM5 mutant is incapable of inducing late viral protein expression. Expression and steady-state levels of all analyzed cellular proteins (β-actin, USP7, Daxx, Rad50, Nbs1, Mre11, and PML) were not altered in UBM2 H5*pm*4250 versus WT H5*pg*4100 infection (Fig. 6A). Comparison of UBM5 H5*pm*4251 to WT H5*pg*4100 surprisingly showed that Daxx was not degraded in UBM5 H5*pm*4251 infections up to 72 hpi (Fig. 6A, lanes 12 to 16).

With WT DBP rescue experiments, we could confirm that the late viral gene expression defect is solely due to the introduced UBM5 mutation (Fig. 5B). To exclude that a certain amount of DBP is required for efficient RC formation and/or the transition from the early to the late phase of infection, we additionally transfected UBM5 H5*pm*4251-infected H1299 cells with a UBM5-encoding plasmid to reach DBP expression levels that are comparable to WT H5*pg*4100 infection. However, we excluded that possibility because even with similar DBP levels, late protein expression was still defective (Fig. 5B).

## DISCUSSION

Ubiquitination is a posttranslational modification that has various effects on target proteins such as the regulation of protein-protein interactions, the intracellular localization of the modified protein, and protein turnover. Consequently, cellular enzymes that regulate ubiquitination play crucial roles in countless cellular pathways. The USP7 deubiquitinase reverses protein ubiquitination, and several groups have reported important proviral, as well as antiviral, roles of USP7 in virus infections (45–50, 69). Interestingly, we previously showed that USP7 colocalizes with DBP (43). Moreover, we demonstrated that USP7 interacts with and stabilizes the multifunctional adenoviral E1B-55K protein and that USP7 had a beneficial impact on both virus replication and cell transformation. Notably, translocation of USP7 into viral RCs and reduced virus replication levels in USP7-depleted cells were independent of the USP7-E1B-55K interaction (43), prompting us to decipher the role of DBP in these processes and to investigate if DBP is a substrate for USP7. Thus, we set out to further explore the impact of USP7 on adenoviral replication and the USP7-DBP colocalization using a panel of DBP mutants with distinct UBM amino acid exchange mutations. We show that DBP interacts with USP7 to regulate viral RC formation and, thus, viral replication. This interaction is facilitated by DBP binding to the USP7 TRAF-like domain through a conserved motif in the N-terminal part of DBP (amino acids 73 to 76 in UBM2) (Fig. 1), a motif that has also been shown to enable USP7 binding of cellular binding partners (65). This is not surprising, as the USP7 TRAF-like domain seems to bind the majority of proteins that interact with USP7 (70–72). However, described motifs in the N-terminal part of DBP only include nuclear localization sequences, as well as phosphorylation- and SUMO-conjugating motifs so far (30, 73), and all other functional DBP domains are within the highly conserved C terminus (42). This work therefore provides important new insights into features of the N- and C-terminal DBP domains. Of all five UBM mutants tested, two exhibited a remarkable phenotype (Fig. 2 and 6), UBM2 (S76A) and UBM5 (S354A). We show that DBP expression and stability and viral DNA replication in UBM2 H5*pm*4250 infections are widely comparable to WT H5*pg*4100 despite the affected recruitment of USP7 into RCs at the 24-hpi time point (Fig. 4). On the other hand, UBM5 binding to USP7 is not altered, but DBP levels decrease with UBM5 H5*pm*4251 (Fig. 2). The fact that UBM5 is highly ubiquitinated indicates that UBM5 proteasomal degradation is accelerated, which likely leads to the observed replication defect (Fig. 3) (74). DBP was found to target PML in infected cells, and interestingly, posttranslational modifications also seem to play a key role in this process, as they regulate DBP and PML interactions at viral RCs (30, 75). Combined, these findings further underline the complexity of posttranslational modifications and their impact on the virus life cycle.

We demonstrate that USP7 and DBP colocalize in viral RCs. Virus-mediated USP7 relocalization has also been described for other viruses and could be a conserved mechanism to promote optimal viral replication conditions (48, 50, 76). Mutational abrogation of the USP7-DBP interaction reveals that it is not essential for USP7 relocalization into viral RCs and is thus dispensable for adenoviral DNA replication (Fig. 4 and 5). Notably, the opposite was observed in MCPyV infection, where USP7 binding to LT and subsequent relocalization to viral RCs negatively regulates viral replication (50).

Unexpectedly, the C-terminal amino acid exchange S354A in UBM5 strongly influences viral late gene expression and completely abrogates viral progeny production (Fig. 5 and 6). Steady-state levels of cellular proteins were not affected in our infections compared to the WT infection, but whether HAdV-C5 RC formation is necessary to degrade Daxx (Fig. 6A) remains to be thoroughly investigated. As a side note, it is worth mentioning that viral E1A steady-state levels maintained at high concentration even at late time points postinfection. It has previously been shown that the degradation of E1A is a prerequisite for the transition to the late stage of infection and a proper HAdV replication cycle (77).

Together, our data clearly show that the UBM5 mutation leads to an increased ubiquitination of the protein, and UBM5 H5*pm*4251-infected cells lack DBP-positive RCs, which are a prerequisite for viral DNA replication and, thus, the transition from the early to the late phase of a productive HAdV infection. Experiments that examine whether the introduced mutations change DBP in a way that it loses its capacity to induce liquid-liquid phase separation and thereby contributes to RC formation are underway. Consequently, though, progeny production is completely abrogated in UBM5 H5*pm*4251-infected cells as a result of a defective DNA replication. This is particularly interesting because it may be an asset for adenoviral vector development.

Adenoviral vectors that can carry a variety of transgenes are successfully used as vaccines. They lack the HAdV E1 region (that is, all E1A and E1B genes) and are therefore replication deficient and require producer cells for virus propagation. To facilitate efficient virus replication in vaccine production settings, the E1 region is stably integrated into the chromosome of these HAdV vector vaccine producer cells (18, 78). This is considered safe but leaves a residual risk of reintroduction of the E1 region into the viral genome by homologous recombination (18, 78, 79) as well as “leaky” virus replication (80, 81). Our UBM5 mutation could act as an additional safety net and further increase vector vaccine safety by ensuring replication deficiency through abrogated RC formation of E2-containing adenoviral vectors harboring the UBM5 mutation. Accordingly, UBM5-modified adenoviruses, propagated in E1- and DBP WT-expressing cell lines, will likely be highly suitable for therapeutic approaches.

In summary, our data provide further evidence on the importance of DBP/E2A for HAdV replication and characterize distinct USP7 binding sites that are crucial for the virus to hijack cellular resources to regulate viral RC formation and produce virus progeny.

## MATERIALS AND METHODS

### Cells.

H1299 cells (ATCC CRL-5803), HCT116 cells (ATCC CCL-247), HEK-293 cells (ATCC CRL-1573), and 2E2 cells (82) were grown and maintained in growth medium comprised of Dulbecco’s modified Eagle’s medium containing 0.11 g/L sodium pyruvate (DMEM; Gibco, Thermo Fisher Scientific) supplemented with 5% to 10% fetal bovine serum (FBS; Pan-Biotech) and 10,000 U/mL penicillin/10 mg/mL streptomycin (Pan-Biotech) in a 5% CO_2_ atmosphere at 37°C. 2E2 cells were used as a helper cell line to propagate DBP mutant viruses. They are derived from 293EBNATet cells that stably express the HAdV-C5 E2 proteins (DNA polymerase, precursor terminal protein, and DBP) and E4orf6 under the control of a tetracycline-dependent promoter (82). 2E2 cells were grown and maintained in growth medium supplemented with 90 μg/mL hygromycin B (Merck Millipore) and 250 μg/mL geneticin (G418; Calbiochem), and expression of the E2 and E4 genes was induced by addition of 1 μg/mL doxycycline, a semisynthetic tetracycline (Thermo Fisher Scientific).

### Plasmids and transient transfections.

The plasmid pE2A-2744 encodes FLAG-tagged WT HAdV-C5 DBP under the control of the cytomegalovirus (CMV) immediate early promoter (30). Plasmids pE2A-3177 (UBM1), pE2A-3178 (UBM2), pE2A-3179 (UBM3), pE2A-3180 (UBM4). and pE2A-3181 (UBM5) were derived from pE2A-2744 by site-directed mutagenesis with oligonucleotide primers (Table 1) changing serine residues at positions 35, 76, 122, 179, and 354 to alanines in the DBP protein. Plasmids expressing Myc-tagged USP7 and His-tagged ubiquitin have been described previously (62, 83). For transient transfections, subconfluent H1299 cells were treated with a transfection mixture of DNA and 25-kDa linear polyethylenimine (PEI; Polysciences). Prior to transfection, the growth medium was removed from the cells and replaced by plain DMEM without fetal calf serum (FCS) and antibiotics. The transfection solution was prepared by incubating a mixture of 1:10:100 (DNA/PEI/DMEM) for 10 min at RT. After application of the transfection solution, cells were incubated for 6 to 8 h in a 5% CO_2_ atmosphere at 37°C before the medium was replaced with growth medium.

**TABLE 1:** Primers used in this study

Primer	Amino acid exchange	Direction	Sequence (5′–3′)
pCMX3b-E2A-UBM1	S35A	Forward	CGTGTCGTCCCCGTCCCCGGCGCCGCCGCCTCCCCGGGC
		Reverse	GCCCGGGGAGGCGGCGGCGCCGGGGACGGGGACGACACG
pCMX3b-E2A-UBM2 and H5*pm*4250 (UBM2)	S76A	Forward	CCAGCCCGCGGCCATCGACCGCGGCGGCGGATTTGGCC
		Reverse	GGCCAAATCCGCCGCCGCGGTCGATGGCCGCGGGCTGG
pCMX3b-E2A-UBM3	S122A	Forward	GCTACAAATGGTGGGTTTCGCCAACCCACCGGTGCTAATC
		Reverse	GATTAGCACCGGTGGGTTGGCGAAACCCACCATTTGTAGC
pCMX3b-E2A-UBM4	S179A	Forward	GCTGAGTGTGCCGATCGTGGCTGCGTGGGAGAAGGGCATG
		Reverse	CATGCCCTTCTCCCACGCAGCCACGATCGGCACACTCAGC
pCMX3b-E2A-UBM5 and H5*pm*4251 (UBM5)	S354A	Forward	CCAATCAGTTTTCCGGCAAGGCTTGCGGCATGTTCTTCTC
		Reverse	GAGAAGAACATGCCGCAAGCCTTGCCGGAAAACTGATTGG
β2 microglobulin (qPCR)		Forward	TGAGTATGCCTGCCGTGTGA
		Reverse	ACTCATACACAACTTTCAGCAGCTTAC
E1B (qPCR)		Forward	GACAGGGCCTCTCAGATGCT
		Reverse	TGGCTACGTGAATGGTCTTCAG
Hexon (qPCR)		Forward	CGCTGGACATGACTTTTGAG
		Reverse	GAACGGTGTGCGCAGGTA

### Viruses.

Viruses were propagated in HEK293, H1299, or 2E2 monolayer cultures. H5*pg*4100 served as the WT HAdV-C5 parental virus in these studies (84). The DBP mutant viruses H5*pm*4250 (UBM2) and H5*pm*4251 (UBM5) were generated and analyzed exactly as described (84). Briefly, point mutations were first introduced into the DBP gene in the L4-Box of pE2-1513 (30) by site-directed mutagenesis using oligonucleotides primers listed in Table 1, resulting in pE2-3197 (UBM2) and pE2-3200 (UBM5). The L4-Box fragment comprising the nucleotides 21438 to 27081 from pH5*pg*4100 was then replaced with the corresponding fragments from plasmids pE2-3197 and pE2-3200 by SgfI/SpeI digestion and subsequent ligation to generate adenoviral plasmids pH5*pm*3224 and pH5*pm*3225, respectively. Finally, the viral genomes were released from the recombinant plasmids by PacI digestion, and the mutant viruses H5*pm*4250 and H5*pm*4251 were generated as described previously (84). Viral genomes from infected cell lysates were sequenced by Sanger sequencing of the DBP regions and next-generation sequencing to confirm the mutations in the DBP gene and verify integrity of the viral genomes.

### Antibodies.

Primary antibodies specific for adenoviral proteins included anti-DBP mouse monoclonal antibody (MAb) B6-8 (85), α-E1A mouse MAb M73 (86), α-E1B-55K mouse MAb 2A6 (87), α-E4orf4 rabbit polyclonal antibody (pAb) (88), α-E4orf6 mouse MAb RSA3 (89), α-L4-100K rat MAb 6B10 (90), and α-capsid protein rabbit pAb L133 (91). Primary antibodies for the detection of cellular and ectopically expressed proteins included α-Daxx rabbit pAb (Upstate), α-Rad50 mouse MAb (GeneTex), α-Nbs1 mouse MAb (Biozol), α-Mre11 rabbit pAb (Novus), α-PML rabbit pAb (Novus), α-β-actin mouse MAb (Sigma-Aldrich), α-FLAG mouse MAb M2 (Sigma-Aldrich), α-histidine (His-tag) mouse MAb (Clontech), and α-USP7 rat MAb 3D8 (43). Secondary antibodies conjugated to horseradish peroxidase (HRP) for detection of proteins by immunoblotting were α-mouse IgG, α-rabbit IgG, and α-rat IgG (Jackson ImmunoResearch).

### Protein analysis and immunoprecipitation.

Cell pellets of transfected or infected cells were lysed in radioimmunoprecipitation assay (RIPA) lysis buffer (50 mM Tris-HCl, pH 7.6, 150 mM NaCl, 5 mM EDTA, 1% [vol/vol] NP-40, 0.1% [wt/vol] SDS, and 0.5% [wt/vol] sodium deoxycholate) on ice for 30 min. The cell lysates were sonicated and subsequently centrifuged to pellet the cell debris (13,000 rpm, 3 min, 4°C). Protein concentrations were determined photometrically using Bradford reagent (Bio-Rad). The same samples were used for immunoprecipitation, Ni-NTA (nitrilotriacetic acid) pulldown, and GST pulldown analyses. To investigate protein-protein interactions, proteins were immunoprecipitated. Here, FLAG-M2-coupled protein A-Sepharose beads (Sigma-Aldrich) were used, or protein A-Sepharose (3 mg/sample) was coupled with 1 μg of the respective antibody for 1 h at 4°C. The antibody-coupled protein A-Sepharose was added to pansorbin-Sepharose (50 μL/lysate; Calbiochem)-precleared extracts and rotated overnight at 4°C. Proteins bound to the antibody-coupled protein A-Sepharose were precipitated by centrifugation and washed three times. Aliquots of the RIPA cell lysates were saved to serve as immunoprecipitation, GST pulldown, and Ni-NTA pulldown input controls. These samples, as well as the protein samples for immunoblotting, were boiled for 3 min at 95°C in Laemmli buffer. Next, the protein samples were separated by SDS-PAGE and visualized by immunoblotting as described previously (92).

### Expression and purification of recombinant fusion proteins (GST pulldown).

The glutathione *S*-transferase (GST) bacterial expression vectors (pGEX; PL-Pharmacia) were designed as follows (see Fig. 1C): GST-USP7-TD (amino acids [aa] 1 to 215) (62), GST-USP7-CD (aa 212 to 561), GST-USP7-C1 (aa 561 to 916), and GST-USP7-C2 (aa 913 to 1102). Expression of GST-fusion proteins in Escherichia coli was induced for 4 h by adding IPTG (isopropyl-β-d-thiogalactopyranoside; VWR) to a final concentration of 1 mM. The bacterial cells were centrifuged (10 min, 6,000 rpm), and the cell pellets were resuspended in MTTB lysis buffer (50 mM Tris, 150 mM NaCl, 1% [vol/vol] Triton X-100, 3 mg/mL lysozyme, 10 U/mL aprotinin, 1 μg/μL leupeptin, and 1 μg/μL pepstatin). After sonication and centrifugation, the supernatant was transferred to a new tube, and 100 μL glutathione Sepharose 4B beads (GE Healthcare) prewashed with MTTB buffer (without lysozyme) was added. The mixture was rotated overnight at 4°C before the beads were pelleted and washed five times with MTTB buffer (without lysozyme). To analyze the protein content, the beads were boiled in Laemmli buffer and analyzed by SDS-PAGE. Proteins were visualized by Coomassie staining. To proof protein-protein interaction with GST pulldown analysis, 800 μg of cell lysate was incubated with the purified beads at 4°C overnight. The samples were washed three times with RIPA buffer, centrifuged, and mixed with Laemmli buffer. After denaturation for 5 min at 95°C, the samples were analyzed by immunoblotting as described above.

### Purification of His-tagged ubiquitin conjugates (Ni-NTA pulldown).

H1299 cells were transfected with plasmids expressing His-tagged ubiquitin and, in some experiments, infected 6 h posttransfection (hpt). At 44 hpt, the medium was changed to plain DMEM without any additives, and the cells were treated with the proteasome inhibitor MG132 (final concentration, 25 μM; Merck). Four hours after the treatment, cells were washed with phosphate-buffered saline (PBS) and subjected to cell lysate preparation and subsequent Ni-NTA purification exactly as described previously (93). All eluates were analyzed by immunoblotting as described above.

### Analysis of viral DNA synthesis by PCR.

Adenoviral DNA replication was determined by conventional PCR. Infected cells were harvested, pelleted, and lysed in RIPA buffer at indicated time points as described above. The cell lysates were treated with Tween 20 (Applichem) and proteinase K (final concentration, 100 μg/mL; Roche) in nucleic acid-free water (Promega) for 1 h at 55°C prior to proteinase K inactivation for 10 min at 100°C. Levels of the adenoviral E1B gene were determined by PCR with E1B-specific oligonucleotide primers amplifying a 389-bp E1B fragment (Table 1). PCR products were visualized by ethidium bromide staining in 1% agarose gels.

### Isolation and quantification of nucleic acids.

Viral and cellular DNAs were isolated from virus stocks and cell pellets according to the QIAamp DNA minikit manual (Qiagen). The DNA samples were quantified by quantitative PCR (qPCR) using a Rotor-Gene 6000 (Corbett Life Sciences, Qiagen). The E1B- and hexon-specific oligonucleotide primers are listed in Table 1. Genomic viral DNA levels were normalized to cellular β2-microglobulin DNA levels.

### Indirect immunofluorescence.

For indirect immunofluorescence, 1 × 10^5^ adherent, eukaryotic cells were seeded on sterile glass coverslips positioned in 6-well cell culture dishes. Twenty-four hours later, cells were transfected or infected with a multiplicity of infection (MOI) of 10 and fixed with paraformaldehyde (PFA; 4% [vol/vol] in PBS) at room temperature (RT) for 20 min at different time points postinfection. The cells were incubated with ammonium chloride (25 mM) at RT for 10 min and permeabilized with Triton X-100 (0.5% [vol/vol] in PBS) at RT for 10 min prior to blocking in Tris-buffered saline-BG (TBS-BG; BG represents 5% [wt/vol] BSA and 5% [wt/vol] glycine) at RT for 10 min. Coverslips were incubated in a humidity chamber for 1 h at RT with the indicated primary antibody diluted in PBS. Afterward, the cells were incubated with the corresponding secondary antibody diluted in PBS (Alexa 488 [Invitrogen]- or Texas Red [Jackson]-conjugated secondary antibodies) for 30 min at RT. Finally, nuclei were stained with DAPI (4,6-diamidino-2-phenylindole) in PBS (1:1,000 [vol/vol] from 1 mg/mL stock) for 5 min before the cells were mounted in glow medium. All steps were separated by three 5 min washing steps with PBS. DAPI was rinsed off with double-distilled water. Digital images were acquired with a widefield fluorescence microscope (Leica) using the Leica Application Suite.

### Statistical analyses.

All statistical analyses were performed with GraphPad Prism v9 (GraphPad Software). Specific information on the statistical tests is provided in the respective figure legends. Data were considered significantly different if the *P* value was ≤0.05.
