# Supplementary material for: A Single Amino Acid Switch in the Adenoviral DNA Binding Protein Abrogates Replication Center Formation and Productive Viral Infection
Source: mBio. 2022 Mar 7;13(2):e00144-22. doi: 10.1128/mbio.00144-22 (PMC9040859; doi:10.1128/mbio.00144-22)
Supplement: FIG S1 [file mbio.00144-22-sf001.pdf]

Supplementary Information for

A single amino acid switch in the adenoviral DNA binding protein abrogates replication center formation and productive viral infection

By Jana Boddin, Wing-Hang Ip, Britta Wilkens, Konstantin von Stromberg, Wilhelm Ching, Emre Koyuncu, Luca D. Bertzbach and Thomas Dobner

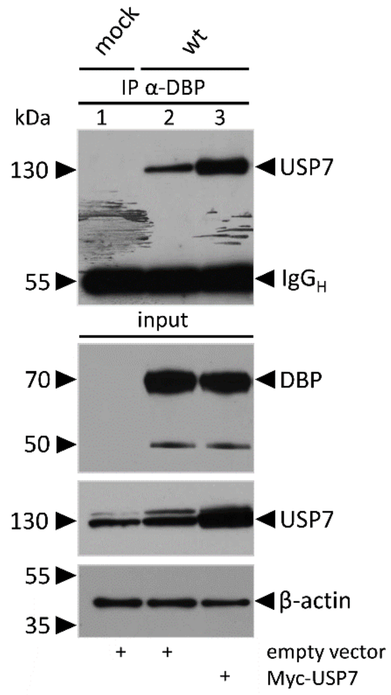

Figure S1: Endogenous USP7 binds DBP in virus-infected HCT116 cells. Empty vector- or Myc-USP7-transfected HCT116 cells were mock-infected or wt HAdV-C5 (H5*pg*4100)-infected 24 h p.t. at an MOI of 20 FFU per cell and harvested 48 h later. Total cell lysates were prepared, DBP was immunoprecipitated using an α-DBP antibody, proteins were resolved by 10% SDS-PAGE and visualized by immunoblotting. Co-precipitated proteins and total cell lysates (input) were analyzed using an α-USP7 antibody. An α-DBP antibody was used to stain DBP in total cell lysates and β-actin served as a loading control. Molecular weights (in kDa) are indicated left and detected proteins right of the blots. Detailed antibody descriptions can be found in the respective materials and methods paragraph.
